# A Multimodal Approach to Managing Severe Psoriasis Vulgaris: A Case Report Leveraging Natural Therapies for Flare Control

**DOI:** 10.3390/life15081186

**Published:** 2025-07-25

**Authors:** Ada Radu, Tunde Jurca, Andrei-Flavius Radu, Teodora Maria Bodog, Ruxandra Florina Bodog, Laura Endres

**Affiliations:** 1 Doctoral School of Biological and Biomedical Sciences, University of Oradea, 410087 Oradea, Romania; adaradu@uoradea.ro (A.R.); bodog.teodoramaria@student.uoradea.ro (T.M.B.); bodog.ruxandraflorina@student.uoradea.ro (R.F.B.); lendres@uoradea.ro (L.E.); 2 Department of Pharmacy, Faculty of Medicine and Pharmacy, University of Oradea, 410028 Oradea, Romania; 3 Department of Psycho-Neuroscience and Recovery, Faculty of Medicine and Pharmacy, University of Oradea, 410073 Oradea, Romania; 4 Department of Surgical Disciplines, Faculty of Medicine and Pharmacy, University of Oradea, 410073 Oradea, Romania

**Keywords:** psoriasis vulgaris, plaque psoriasis, natural bioactive compounds, Dead Sea treatments, inflammation, diet

## Abstract

A psoriasis vulgaris flare is characterized by a rapid intensification of symptoms, which is often triggered by various factors that can worsen the condition. The risk factors for these exacerbations are numerous and include obesity, antihypertensive drugs, and psychological stress. Moreover, links have been documented between type II diabetes, hypertension, and psoriasis vulgaris. The present case report describes a 52-year-old female patient who presented at the clinic with disseminated erythematous-squamous plaques and patches covered by thick, white-pearly, easily detachable scales, along with stress, fatigue, anxiety, severe pruritus, irritability, insomnia, and decreased self-esteem. Her past medical regimen included various conventional topical options, including calcipotriol combined with betamethasone, clobetasol, betamethasone combined with salicylic acid, and betamethasone combined with gentamicin, yet the condition remained refractory, with periodic flare-ups. The integrated and personalized therapeutic approach aimed to target both the dermatological issues and the associated systemic and psychological factors contributing to the condition. The therapeutic strategy implemented in this case combined psychological counseling sessions, a very low-calorie ketogenic diet, oral supplementation with anti-inflammatory and antioxidant vitamins and minerals, topical treatments utilizing urea and Dead Sea-mineral-based formulations, and rosemary extract-based scalp care, without requiring additional conventional treatment. This comprehensive approach led to significant improvement, ultimately achieving complete remission of the patient’s psoriasis. The associated comorbidities were well controlled with the specified medication, without any further complications. Thus, the importance of alternative options was emphasized, particularly in the context of an incurable disease, along with the need for continued research to improve the ongoing therapeutic management of psoriasis vulgaris. Such approaches are essential to reducing the risk of flare-ups and to achieving better management of associated risk factors.

## 1. Introduction

Psoriasis is a chronic autoimmune skin condition characterized by a recurring course with flare-ups and periods of remission. This disorder can develop at any stage of life, though its incidence tends to follow a bimodal pattern. It typically first manifests in individuals between the ages of 20 and 30, with a second peak around 50 to 60 years. The disease can severely impact a person’s quality of life, as many affected individuals experience not only physical discomfort but also significant emotional and social distress [1,2].

Psoriasis vulgaris, commonly referred to as plaque psoriasis, represents the predominant clinical manifestation, accounting for almost 90% of all psoriasis cases. It is characterized by well-demarcated erythematous plaques covered by silvery-white scales, which are typically symmetrically distributed on the knees, elbows, scalp, and sacral region. Its diagnosis is primarily clinical, with biopsy indicated only in atypical or uncertain cases [2,3,4].

To assess the disease’s severity and its impact, several standardized instruments are commonly used, such as the Psoriasis Area and Severity Index (PASI), the body surface area percentage, and the Dermatology Life Quality Index (DLQI). Despite their widespread use, none of these scoring systems fully satisfy the comprehensive validation requirements for an optimal assessment tool [5].

Psoriasis vulgaris is an immune-mediated condition with a complex etiology involving both genetic predisposition and environmental triggers [6]. Although keratinocyte dysfunction contributes to epidermal hyperproliferation, the primary driver is immune dysregulation. T-cell-mediated inflammation is central, beginning with dendritic cell and macrophage activation, which promotes interleukin (IL)-23 production and Th17 cell expansion. Th17 lymphocytes secrete IL-17 and IL-22, stimulating keratinocyte activation, epidermal thickening, and dermal immune cell infiltration. In parallel, Th1 and natural killer cells release interferon-gamma and tumor necrosis factor-alpha (TNF-α), sustaining chronic inflammation. TNF-α enhances leukocyte recruitment and maintains IL-6 and IL-8 production, with IL-8 promoting neutrophil accumulation and angiogenesis. An imbalance between Th17 and Treg cells, including Treg dysfunction and plasticity toward IL-17-producing phenotypes, sustains a self-amplifying inflammatory loop. These immune-driven mechanisms form the core of psoriasis pathogenesis, which provides a rationale for therapies that modulate specific components of the immune response [7].

Psoriasis vulgaris can be managed with a variety of treatment options, including biologics like TNF-α inhibitors (e.g., adalimumab), IL-23 inhibitors (e.g., guselkumab), and IL-17 inhibitors (e.g., secukinumab). Other alternatives such as ustekinumab and Janus kinase inhibitors (e.g., tofacitinib), as well as novel therapies like piclidenoson, belumosudil, cedirogant, and apremilast, offer additional flexibility. Furthermore, therapeutic options can be tailored according to the severity of the condition and the patient’s needs [8].

Topical treatments are the cornerstone in managing psoriasis vulgaris, primarily due to their favorable safety profile. These include tazarotene, calcineurin inhibitors like tacrolimus and pimecrolimus, corticosteroids, vitamin D analogues, salicylic acid, coal tar, anthralin, and moisturizers. Their minimal side effects make them a preferred option in therapy [9]. The use of plant-derived compounds and other natural resources has led to the rise of phyto-pharmaceuticals, which are increasingly being recognized as adjunctive and alternative therapies for managing the symptoms of psoriasis vulgaris [10].

However, despite the breadth of pharmacological options, therapeutic resistance or reduced efficacy over time may occur in a subset of patients. There are cases in which patients exhibit an inadequate response or experience a loss of efficacy of biologic treatments, which leads to disease flare-ups and the emergence of refractory disease [11]. As a result, interest is growing in complementary therapeutic strategies, particularly those derived from natural sources, due to their potential to reduce inflammation and oxidative stress with fewer side effects [12].

Dead Sea mud and minerals have shown promising benefits in dermatology due to their anti-inflammatory and skin-repairing properties. Rich in essential elements, they may support the healing of irritated skin and improve hydration. Their potential for use in chronic skin disorders such as psoriasis lies in their ability to modulate inflammation and promote tissue repair [13].

An optimal and holistic approach to managing psoriasis vulgaris involves also addressing lifestyle factors such as stress and diet. Promising results have been observed with specific dietary strategies, including the Mediterranean diet, which focuses on fresh foods and polyunsaturated fatty acids, gluten-free regimens for individuals with celiac disease, and calorie-restricted plans aimed at weight reduction in patients who are obese [14]. Furthermore, the relationship between psoriasis and stress is intricate, with stress acting as both a potential trigger and a result of psoriasis flare-ups. Research indicates that individuals who have experienced stressful events within the past year are more likely to develop psoriasis, which implies that stress may contribute to disease onset in those who are genetically predisposed. Recognizing the impact of stress on psoriasis underscores the importance of incorporating stress management strategies when devising treatment plans for affected patients [15].

The present case report aims to emphasize the importance of managing refractory forms of psoriasis vulgaris by using complementary therapeutic options with anti-inflammatory and antioxidant effects that are integrated in a personalized treatment plan that targets the axis of topical natural source therapies–dietary strategies–stress management approaches. By combining these strategies, optimal results were obtained, and a more effective control of psoriatic symptoms was maintained in this case of psoriasis vulgaris that was resistant to conventional topical therapy. The scientific contribution of this study is based on the distinct proposal of an integrated approach that takes into account the importance of searching for alternative solutions with minimal adverse effects, as well as the awareness and management of lifestyle factors that may influence the evolution and severity of psoriasis vulgaris.

## 2. Detailed Case Description

A 52-year-old female patient from an urban area presented at the clinic with extensive, disseminated erythematous-squamous plaques and patches with thick, white-pearly scales that were easily detachable that involved over 30% of her body surface area. These were accompanied by severe pruritus, intense fatigue, insomnia, irritability, anxiety, and decreased self-esteem, all significantly impairing her daily functioning and psychosocial well-being.

The patient reported that the initial manifestation of psoriasis occurred approximately three decades ago, beginning with localized plaques that were predominantly on the extensor surfaces of the elbows and knees. Over time, the disease evolved into a generalized form, progressively spreading to involve the trunk, upper and lower limbs, and scalp.

The patient reported a chronic relapsing-remitting course, with periods of exacerbation and partial remissions, noting that the flare-ups were often preceded or exacerbated by identifiable triggers including intense occupational stress, familial emotional distress, and environmental factors such as seasonal changes or infections. Her medical history was significant for multiple cardiometabolic comorbidities, including essential hypertension, type II diabetes mellitus, and class I obesity, all of which contribute to a pro-inflammatory systemic state known to aggravate psoriasis. She was treated for hypertension with 5 mg of bisoprolol fumarate per day, 5 mg perindopril arginine per day, and 1.5 mg of indapamide 1.5 mg per day in a sustained-release format and for diabetes with 1000 mg of metformin hydrochloride per day.

Despite undergoing various treatments, including topical agents in semisolid pharmaceutical forms that contained calcipotriol combined with betamethasone, clobetasol, betamethasone combined with salicylic acid, and betamethasone combined with gentamicin, alongside homeopathic remedies and active balm, the disease remained refractory with no sustained remission and no clinically meaningful improvement (Table 1).

Throughout the clinical course, the patient demonstrated adherence to the therapeutic protocols prescribed by her attending dermatologist, systematically progressing through established topical treatment regimens. Nevertheless, systemic or biologic interventions had not been initiated, which was attributed to either patient preferences or clinical considerations regarding comorbid conditions and the patient’s suboptimal response to previous topical therapies. At this point, the patient expressed a strong preference for implementing comprehensive lifestyle modifications and addressing psychological factors, rather than just pursuing conventional systemic interventions. This clinical trajectory underscores the refractory nature of her psoriatic condition and emphasizes the imperative for implementing a comprehensive, multidisciplinary therapeutic approach.

**Figure 1:**
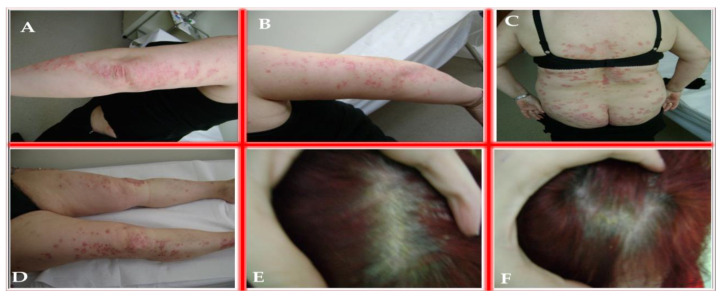
Generalized form of psoriasis vulgaris. Erythematous-squamous plaques and patches with thick, white-pearly scales that are easily detachable, observed on the: (**A**) elbows; (**B**) arms; (**C**) chest; (**D**) knees and legs; (**E**,**F**) scalp.

The primary diagnosis for the patient was psoriasis vulgaris in a generalized form. Upon physical examination, numerous well-demarcated, erythematous plaques with thick, silvery-white scales were noted on multiple body regions including the bilateral elbows, arms, chest, knees, legs, and scalp. Nail examination revealed pitting, onycholysis, and subungual hyperkeratosis, particularly affecting the fingernails. The skin surrounding the plaques was dry and xerotic, with mild secondary excoriations. The extent and severity of the lesions are depicted in Figure 1. Furthermore, differential diagnoses considered during the evaluation included psoriasiform drug eruptions, lichen simplex chronicus, tinea corporis, and mycosis fungoides.

Disease severity indices were markedly elevated, reflecting the profound disease burden: the DLQI score was 30 (indicating very severe impact), the PASI score was 39 (severe disease), the Nail Psoriasis Severity Index score was 46, and the Psoriasis Scalp Severity Index score was 30. These scores correlate with significant impairment in physical comfort, psychological health, and social interactions.

Laboratory investigations revealed a dysmetabolic profile (i.e., elevated serum levels of cholesterol, glucose, and triglycerides), which was accompanied by a reduced serum iron concentration that was likely secondary to chronic inflammation or dietary insufficiency. The erythrocyte sedimentation rate (ESR) was mildly elevated, suggesting ongoing systemic inflammation, whereas the fibrinogen and C-reactive protein (CRP) levels remained within normal limits, possibly reflecting the chronic, low-grade inflammatory nature of psoriasis in this patient (Table 2).

Abdominal ultrasonography revealed grade I hepatic steatosis, which was consistent with the patient’s metabolic dysregulations. The patient’s BMI was measured at 30.9 kg/m^2^, which indicates class I obesity, a known risk factor for psoriasis severity and treatment resistance.

The histopathological examination of a lesional skin biopsy confirmed the diagnosis of psoriasis vulgaris, showing epidermal hyperplasia with marked acanthosis, elongated rete ridges, papillomatosis, hyperparakeratosis, and collections of neutrophils within the stratum corneum forming Munro microabscesses. Additionally, the papillary dermis exhibited vascular ectasia along with a chronic inflammatory infiltrate, which is consistent with the typical histological profile of psoriatic lesion. The biopsy also revealed epidermal hyperplasia with keratinocyte proliferation and inflammatory cell infiltration that corroborated the clinical severity that was observed. These histological features confirmed the active inflammatory status of the disease and supported the need for a comprehensive therapeutic strategy.

**Figure 2:**
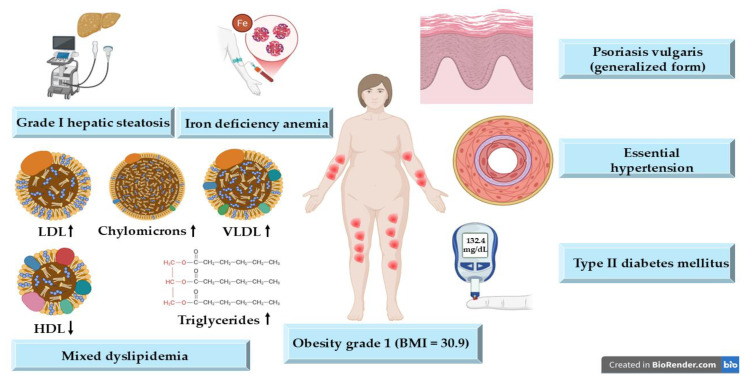
Multimorbidity profile with a generalized form of psoriasis vulgaris and metabolic-comorbid overlap. BMI, body mass index; LDL, low-density lipoprotein; HDL, high-density lipoprotein; VLDL, very low-density lipoprotein; ↓, decrease; ↑, increase.

Figure 2 presents the patient’s overall pathological profile after the anamnesis, objective examination, clinical examination, and paraclinical examination, highlighting the presence of multiple comorbidities that contribute to a highly complex and challenging therapeutic management plan.

A comprehensive, multidisciplinary therapeutic strategy was implemented with the aim of addressing both the cutaneous manifestations of psoriasis vulgaris and the patient’s lifestyle, while also targeting psychological comorbidities, in a synergistic and individualized manner. This approach included tailored natural dermatological care (i.e., day cream, night cream, shampoo every 2 days), lifestyle and dietary adjustments, supportive supplementation, stress management, and a structured psychotherapeutic intervention plan (i.e., weekly for 3 months). Specifically, the topical regimen consisted of the use of emollients that contained 3–10% urea to improve the patient’s skin hydration and reduce scaling, two creams enriched with Dead Sea minerals (day cream with chamomile extract, olive oil, linoleic acid; night cream with cocoa butter, lanolin, Dead Sea mud, thymol), and a rosemary-extract-based shampoo that was applied every two days to control scalp involvement.

The general supportive measures included oral daily supplementation with fat-soluble vitamins A (5000 IU/day), D2 (ergocalciferol 1000–2000 IU/day), and vitamin E (400 IU/day), all of which were administered daily to help modulate the patient’s immune responses and support their skin barrier function. Additionally, a multimineral complex was introduced, containing calcium (500–1000 mg/day), magnesium (200–400 mg/day), phosphorus (700 mg/day), potassium (100–200 mg/day), zinc (15–30 mg/day), iron (as needed based on serum iron levels, typically 14–18 mg/day), copper (1–2 mg/day), manganese (1–3 mg/day), and iodine (150 µg/day), that was tailored to address potential deficiencies and support the patient’s systemic metabolic balance.

Passiflora incarnata extract (200–400 mg twice daily) was prescribed as a phytotherapeutic agent to reduce the patient’s stress and improve their sleep quality, complementing weekly psychological counseling based on cognitive-behavioral therapy techniques to reduce the patient’s anxiety, improve their sleep quality, and enhance their emotional resilience, all of which are critical for psoriasis control.

The patient was also advised on and successfully adhered to a medically supervised very low-calorie ketogenic diet (VLCKD), which contributed to weight loss and a reduction in systemic inflammatory markers, and which was monitored monthly by clinical and laboratory evaluations.

The associated comorbidities were well controlled with the specified medication, with only transient exacerbations and elevations in biochemical parameters being observed during flare-up periods, and no additional complications.

**Figure 3:**
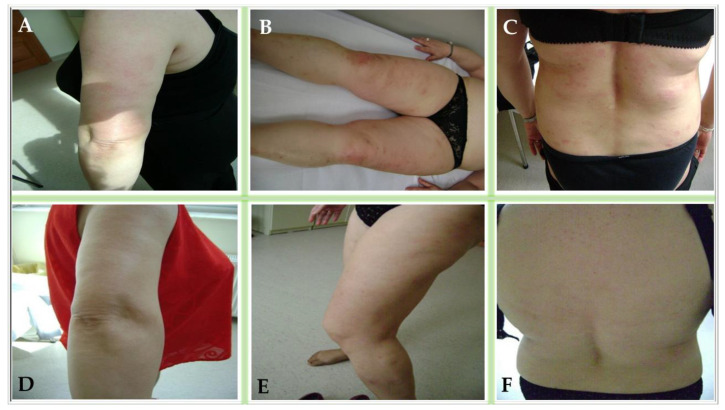
Clinical progression and therapeutic response over time. Mild erythematous plaques and patches without scaling observed at one month on the elbows and upper limbs (**A**), knees and lower legs (**B**), and posterior trunk (**C**). Complete clearance of lesions documented at three months on the elbows and upper limbs (**D**), knees and lower legs (**E**), and posterior trunk (**F**).

The patient demonstrated a marked response to therapy. Within one-month, psoriatic plaques exhibited the complete resolution of surface scaling, with only minimal residual erythema being noted on clinical examination (Figure 3A–C). This clinical improvement was paralleled by a progressive reduction in severity scores. At 1 month, follow-up laboratory tests showed a decrease in the serum glucose by 12%, total cholesterol by 15%, and triglycerides by 18%, as well as normalization of the ESR, indicating a positive systemic anti-inflammatory effect. The patient’s BMI decreased to 29.8 kg/m^2^.

By three months, the patient had achieved full clearance of skin lesions, which was accompanied by substantial enhancement in their psychological well-being and the normalization of all composite indices (Figure 3D–F).

At 3 months, the laboratory parameters further improved: the glucose decreased by 20% from baseline, the lipid profile normalized, the ESR remained within normal limits, and the BMI reached 28.5 kg/m^2^, demonstrating sustained metabolic and clinical benefits.

Over time, the patient experienced very rare disease flares, which were notably milder and less frequent than the initial presentation. The management of these episodes consisted only of the ongoing application of urea-based emollients, topical formulations containing Dead Sea-derived minerals, and the implementation of individualized stress reduction techniques and dietary strategies. Sustained adherence to a structured dietary plan and regular psychological support further contributed to maintaining disease stability. The patient exhibited excellent adherence to the therapeutic regimen, a factor that was deemed essential for sustaining long-term remission.

This study was conducted with the approval of The Ethics Committee of the Oradea Pelican Clinical Hospital, Romania, under reference number 12/05.01.2018, and in accordance with the ethical principles outlined in the World Medical Association Declaration of Helsinki for medical research involving human participants [22]. Written informed consent was obtained from the patient for the presentation of the present clinical case.

## 3. Discussion

The most frequently encountered form of psoriasis in clinical practice is psoriasis vulgaris. This condition is often linked to systemic dysfunctions beyond the skin. A significant number of affected individuals experience periodic exacerbations, with episodes that may intensify and, in certain cases, evolve into more aggressive variants such as pustular psoriasis [23].

Epidemiological data suggest a bimodal pattern regarding the initial manifestation of psoriasis. The first incidence peak typically occurs in early adulthood, around the age of 22, which characterizes the early-onset form of the disease. A second, later peak is generally observed after the age of 50, marking the onset of late-onset psoriasis. The patient described in this case aligns with the early-onset subgroup, with the disease onset having occurred at approximately 25 years of age. Furthermore, the clinical features are consistent with the classic presentation of psoriasis vulgaris, which is characterized by erythematous plaques and patches covered with thick, white-pearly scaling [24].

Multiple precipitating elements have been implicated in the exacerbation of psoriatic disease. Among these, microbial imbalances at the level of the gut and skin, infectious agents, unfavorable lifestyle habits, specific pharmacological agents, and psychological distress play significant roles in triggering disease flares [25]. In this particular case, potential contributing factors to the disease’s exacerbation include the patient’s class I obesity (BMI 30.9), along with their use of pharmacological agents such as the beta-blocker bisoprolol fumarate and the ACE inhibitor perindopril arginine, which is used for the management of hypertension.

In addition, the patient’s reported experience of ongoing occupational and family-related stress, along with underlying anxiety, may contribute to the dysregulation of their hypothalamic–pituitary–adrenal axis, a mechanism recognized for its role in triggering psoriasis flares. The high prevalence of anxiety and depressive symptoms among individuals with psoriasis likely reflects a dual influence—both from systemic inflammatory mediators and from the psychosocial burden of living with a chronic dermatologic condition. These observations underscore the critical need for the use of psychological support and stress-reduction strategies as integral components of effective disease management [26].

A wide range of therapeutic strategies exist for addressing psoriasis vulgaris (chronic plaque-type psoriasis), encompassing topical formulations such as corticosteroids and vitamin D3 derivatives, ultraviolet light-based interventions, systemic pharmacologic agents, and targeted biologic treatments like methotrexate, etanercept, and adalimumab. Although numerous therapeutic avenues are accessible, a notable subset of patients fails to attain full and lasting remission, frequently continuing to struggle with diminished quality of life. The sustained control of psoriasis presents considerable difficulty, primarily due to its cyclical, relapsing pattern, alongside commonly encountered issues such as delayed therapeutic efficacy and suboptimal treatment adherence [27,28]. Nevertheless, for a significant proportion of individuals affected by this condition, topical therapies alone remain sufficient for disease control [27].

Being a refractory psoriatic form that has been managed over time with topical treatments such as calcipotriol, salicylic acid, corticosteroids and antibiotics, this case has highlighted in an integrated manner the importance of a complex, alternative, and natural management approach that targets vitamin and mineral supplementation and the application of urea and minerals present in special creams from Dead Sea, with an extra attention and focus on dietary strategies and stress reduction through supplements and psychological counseling.

Data in the scientific literature suggest an association between the use of vitamins A, D, and E [29,30], the use of minerals like calcium, magnesium, and zinc [31,32], and an improved management of psoriasis vulgaris, which was also observed in our patient. Additionally, clinical improvement was observed in a reported case involving a patient experiencing febrile flares of pustular psoriasis in the context of marked hypocalcemia, where calcium replacement therapy by itself proved effective in resolving symptoms [33].

Extensive research has demonstrated a strong link between psoriasis and a heightened incidence of comorbid conditions, including type 2 diabetes mellitus and hypertension. This association is largely attributed to persistent systemic inflammation, which contributes to the development of insulin resistance, vascular endothelial impairment, and increased arterial rigidity [34]. This was also the case for our patient, who developed hypertension and type 2 diabetes following the onset of psoriasis vulgaris. In addition to lifestyle modification recommendations, the patient was advised to consult a cardiologist and an endocrinologist for further assessment of dyslipidemia, cardiovascular risk, and optimal blood glucose control, given the elevated levels observed during the paraclinical evaluation that was integrated into the overall management of their psoriasis vulgaris.

Selenium (Se), an essential trace element, plays a vital role in regulating oxidative stress and inflammation through its incorporation into selenoproteins such as glutathione peroxidase (GPX), selenoprotein-P, and selenoprotein-S. These molecules help maintain immune balance and tissue repair, particularly during skin injury, by neutralizing reactive oxygen species (ROS) and modulating inflammatory cytokines—processes that are highly relevant to chronic skin conditions like psoriasis [35]. In psoriatic patients, the Se levels are significantly lower in the serum and whole blood compared to healthy individuals, with an inverse relationship to disease severity indices such as PASI, NAPSI, and BSA. This deficiency is associated with the impaired activity of antioxidant enzymes like GPX1 and catalase, contributing to heightened oxidative stress, keratinocyte apoptosis, and persistent inflammatory signaling—factors that exacerbate psoriatic lesions. Genetic predispositions that affect Se metabolism may further influence the Se status in psoriasis. Dysregulation in selenoenzyme function, especially in individuals with common metabolic gene variants, can worsen the systemic antioxidant imbalance, promoting psoriatic inflammation and immune dysregulation [35,36].

Meta-analytic evaluations confirm that the Se levels are consistently reduced in people with psoriasis, particularly in blood-based measurements. Although higher concentrations may be found in non-systemic compartments like hair, systemic Se deficiency aligns closely with disease presence and progression, which underlines its pathophysiological significance [37]. Furthermore, due to the fact that Se is derived from dietary sources and influenced by soil content, geographic and nutritional variability affects individuals’ Se status. Despite its known antioxidant and anti-inflammatory roles, routine Se supplementation remains controversial due to limited and inconsistent clinical trial outcomes in psoriasis populations [38].

Amid the ongoing exploration of novel treatment strategies to enhance psoriasis management, topically applied selenium nanoparticles have emerged as a promising approach, demonstrating therapeutic efficacy by suppressing keratinocyte hyperproliferation and dampening key inflammatory pathways. By generating ROS and inducing apoptosis, selenium nanoparticles downregulate molecular markers like MAPKs, STAT3, cyclin-D1, and Ki67, significantly alleviating psoriatic features such as acanthosis and immune overactivation [39].

The topical approach involved utilizing formulations that contained urea, which have been proven to be effective in improving clinical outcomes in various skin conditions that present with dryness and scaling, such as atopic dermatitis, xerosis, and psoriasis. This treatment strategy was similarly incorporated into the management plan for our patient [40].

While the positive impacts on psoriasis of Dead Sea climatotherapy and bathing are well-documented, as they lead to a marked decrease in most inflammatory skin biomarkers, with the notable exception of CD207 [41,42], the effect of incorporating Dead Sea mud into topical formulations remains less explored and requires further investigation. Research has demonstrated that Dead Sea mud is well-tolerated on the skin and does not compromise the integrity of the skin barrier in healthy individuals [43]. Additionally, Dead Sea cosmetics have demonstrated anti-inflammatory, skin barrier-repairing, and skin-moisturizing properties [44], characteristics that were desired and successfully applied in the present case, where Dead Sea mud was contained in the applied night cream together with cocoa butter, lanolin, and thymol.

According to research, *Rosmarinus officinalis* has antioxidant qualities that could assist in healing viral and inflammatory dermatological conditions. These results align with the approach in the current case, where a rosemary-extract-based shampoo was recommended for managing scalp involvement [45]. *Rosmarinus officinalis* L., commonly known as rosemary, is a medicinal plant that is traditionally used for treating a wide array of conditions, particularly inflammatory, neurological, digestive, and metabolic disorders. Its complex phytochemical profile includes 49 known compounds from six distinct classes, with rosmarinic acid and caffeic acid being the predominant phenolic constituents [46].

The pharmacological potential of rosemary arises from its synergistic metabolites, including carnosic acid, carnosol, and rosmarinic acid, which contribute to its antioxidant, anti-inflammatory, neuroprotective, antimicrobial, anticancer, and hepatoprotective actions [47]. Among these, carnosol and carnosic acid demonstrate significant activity against oxidative stress by scavenging peroxyl and hydroxyl radicals, inhibiting lipid peroxidation more effectively than synthetic antioxidants like butylated hydroxytoluene and butylated hydroxyanisole. These compounds also act as metal ion chelators (e.g., Fe^2+^), reducing the formation of reactive oxygen species and stabilizing cellular membranes. During fruiting stages, rosemary exhibits enhanced antioxidant capacity, with increased levels of carnosol, rosmarinic acid, and hesperidin [48].

Anti-inflammatory effects are likewise well-supported. Rosemary extract inhibits proinflammatory cytokines such as TNF-α and IL-1β in lipopolysaccharide-stimulated macrophages without significant cytotoxic or genotoxic effects, even at concentrations that promote >50% cell viability. This supports its potential for safe use in topical formulations targeting local inflammation [49]. Therefore, *Rosmarinus officinalis* L. delivers diverse, functional benefits within skincare and cosmetic industries, supplying botanical alternatives for numerous dermatological issues [50].

The benefits of topical application are further reinforced by studies on ursolic, oleanolic, and micromeric acids, which exhibit strong anti-inflammatory, wound-healing, UV-protective, and antinociceptive effects. Carnosic acid reduces nitric oxide levels, carnosol alleviates atopic dermatitis, and whole rosemary extract has shown anti-inflammatory efficacy comparable to that of indomethacin [51]. Despite promising data, larger clinical trials remain necessary to fully validate rosemary’s dermatological applications [45].

Studies have demonstrated that *Passiflora incarnata* extract significantly reduces stress levels, as measured by the Perceived Stress Scale, while also notably increasing the total sleep time compared to placebo [52]. Based on scientific evidence, it was utilized to manage both occupational and familial stress in the current case, in conjunction with specialized psychological counseling sessions.

A comprehensive review that examined clinical studies on the impact of dietary patterns in managing psoriasis vulgaris concluded that adherence to calorie-restricted, gluten-free, or Mediterranean diets may correlate with positive improvements in psoriasis symptoms [53].

Considering that our patient’s BMI is 30.9, a decision was made to initiate a medically supervised VLCKD. This approach, through ketone body production, has been linked to notable reductions in both body weight and inflammatory markers [54].

The combination of all the recommended interventions resulted in significant improvement after one month, with complete remission being observed by the third month. This highlights the importance of bioactive compounds derived from natural sources, which have shown potential in reducing inflammation and oxidative stress in psoriasis, while causing fewer side effects.

## 4. Conclusions

The integrated therapeutic approach applied in the present case report, combining oral supplementation with anti-inflammatory and antioxidant vitamins and minerals, the topical application of urea and Dead Sea-mineral-enriched formulations, scalp care with rosemary extract, dietary intervention through VLCKD, and structured stress reduction, proved highly effective in achieving complete remission of the generalized form of psoriasis vulgaris. By targeting multiple interconnected factors involved in psoriasis flares, such as systemic inflammation, oxidative stress, metabolic imbalance, and psychological distress, this case underscores the clinical potential of personalized, natural, and multimodal strategies in managing refractory forms of psoriasis vulgaris.

## Figures and Tables

**Table 1:** Topical therapeutic strategies administered over time.

Active Substance	Concentration	Pharmaceutical Form	Mechanism of Action	Reference
Calcipotriol (vitamin D3 analogue)	50 µg	Gel	Binds retinoid X receptor, regulates cell differentiation and immune function	[16]
Betamethasone Dipropionate	0.5 mg	Gel	Inhibition of T-cell activation, reduced production of pro-inflammatory cytokines, induction of apoptosis and inhibition of proliferation	[17]
Clobetasol Propionate	0.5 mg	Cream	Stabilizes cell membranes, modulates immune cells, reduces inflammation and proliferation	[18]
Salicylic acid	20 mg	Cutaneous solution	Keratolytic action	[19]
Gentamicin sulphate	1 mg	Cream	Inhibition of protein synthesis by targeting the 30S ribosomal subunit	[20]
Calcarea carbonica	200 C	Granules	Overall impact on systemic predispositions, inflammation, and skin texture	[21]

C, centesimal.

**Table 2:** Laboratory assessment of relevant metabolic and inflammatory parameters.

Parameter	Value	Reference Range
CRP	0.4 mg/dL	<0.5 mg/dL
ESR	40 mm/h	<30 mm/h
Fibrinogen	340 mg/dL	200–400 mg/dL
Glucose	132.4 mg/dL	60–99 mg/dL
Serum iron	30 µg/dL	34–145 µg/dL
Total cholesterol	280 mg/dL	<200 mg/dL
Triglycerides	185 mg/dL	<150 mg/dL

CRP, C-reactive protein; ESR, erythrocyte sedimentation rate.

## Data Availability

The raw data supporting the conclusions of this article will be made available by the authors on request.

## Abbreviations

The following abbreviations are used in this manuscript:

PASI	Psoriasis Area and Severity Index
DLQI	Dermatology Life Quality Index
TNF- α	Tumor necrosis factor alpha
IL	Interleukin
ESR	Erythrocyte sedimentation rate
CRP	C-reactive protein
VLCKD	Very low-calorie ketogenic diet
BMI	Body mass index
JAK	Janus kinase
GPX	Glutathione peroxidase
ROS	Reactive oxygen species
